# Dairy Cattle Feed Upcycling, Adaptogenic Nutraceuticals and Precision Reproduction: Advanced CASA Sperm Kinematic Profiling and Sexual Drive in Rams

**DOI:** 10.3390/vetsci13101009

**Published:** 2026-09-24

**Authors:** César A. Meza-Herrera, Ángeles De Santiago-Miramontes, Andrés J. Rodríguez-Sánchez, Karla Q. Ramírez-Uranga, Raquel Santos-Silva, Ulises Macías-Cruz, Cayetano Navarrete-Molina, Denia L. Vargas-García, Mayela Rodríguez-González, Edén A. Luna-Zapién, María A. Sariñana-Navarrete

**Affiliations:** 1 Graduate Program—Natural Resources and Environment in Arid Lands, Regional Universitary Unit on Arid Lands, Chapingo Autonomous University, Bermejillo 35230, Durango, Mexico; 2 Graduate Program—Agricultural and Livestock Sciences, Laguna Unit, Antonio Narro Agrarian Autonomous University, Torreon 27054, Coahuila, Mexico; 3 Institute of Agricultural Sciences, Autonomous University of Baja California, Mexicali 21705, Baja California, Mexico; 4 Division of Health, Biological and Environmental Sciences, Open and Distance University of Mexico, Ciudad de México 03330, Mexico; 5 Faculty of Chemical Sciences, Juarez University of the State of Durango, Gomez Palacio 35010, Durango, Mexico; 6 Department of Sustainable and Protected Agriculture, Technological University of Rodeo, Rodeo 35760, Durango, Mexico

**Keywords:** circular economy, upcycled dairy orts, *Withania somnifera*, *Lepidium meyenii*, sperm kinematics, trajectory linearity, sexual drive, semen–sperm quality

## Abstract

This study capitalizes on computer-assisted semen analysis (CASA) to evaluate a precision nutritional strategy integrating agro-industrial feed upcycling and functional phytogenic supplementation in rams. Black Belly rams (n = 15) sustained on an upcycled basal diet derived from dairy-cow feed rejections were assigned to a non-supplemented control group (CONT) or supplemented with *Withania somnifera* (WITH) or *Lepidium meyenii* (LEPI) at 400 mg kg^−1^ LW d^−1^. The 64-day trial was conducted from May to July in arid northern Mexico. Sexual drive, seminal quality, and high-resolution sperm kinematics were evaluated using CASA. Body and testicular measurements, ejaculate volume, and most categorical motility traits remained comparable among treatments (*p* > 0.05). However, phytogenic supplementation enhanced selected seminal and sexual-drive variables (*p* < 0.05). Crucially, longitudinal treatment × time interactions (*p* < 0.05) showed that while the CONT group experienced a seasonal decline in local mobility (LOCMOB), it increased from 1.47% to 2.01% in the WITH group, representing a 37% relative increase over time. Moreover, both WITH and LEPI increased LIN and STR by the end of the experimental period (*p* < 0.05), demonstrating more linear and efficient sperm trajectories. These findings highlight the productive value of upcycled dairy-cow feed rejections and demonstrate that strategic supplementation with *W. somnifera* or *L. meyenii* can refine sexual drive, seminal quality, and sperm kinematic efficiency. This integrated bio-circular strategy provides actionable insights for sustainable small-ruminant production under challenging arid summer conditions.

## Simple Summary

High feed costs and seasonal heat stress severely limit the reproductive efficiency of sheep farms. This study explores an innovative, eco-friendly solution: feeding rams a basal diet based on upcycled feed rejected by large-scale, industrialized dairy cow operations—a strategy defined as “The Robin Hood Effect”—and supplementing them with natural herbal extracts (*Withania somnifera* and *Lepidium meyenii*). Our results demonstrate that the upcycled dairy feed perfectly maintained the rams’ body condition and testicular function. Interestingly, conventional motility differed little among groups, whereas phytogenic supplementation enhanced sexual drive, and computer-assisted sperm analysis (CASA) revealed selective improvements in sperm linearity and straightness. This circular economy farming approach proves that industrial agricultural waste can be successfully repurposed to boost sheep reproduction, even in the summer heat-stress period, lowering production costs while improving the livelihoods of sheep farmers and their families.

## 1. Introduction

From an economic perspective, reproductive efficiency is a fundamental determinant for optimizing pregnancy rates and reducing the generation interval within a livestock enterprise [1]. Consequently, male reproductive soundness is central to maximizing herd viability, genetic progress, and economic returns [1,2]. While the testes exert critical endocrine and exocrine control via testosterone production and spermatogenesis, respectively, their overall functional capacity is heavily modulated by nutritional plane [2,3,4]. Testicular biometrics exhibit a direct correlation with overall functional capacity, as approximately 98% of the testicular mass is composed of seminiferous tubules and germ cells [4], both of which are highly sensitive to nutritional variations [2,3]. In northern Mexico’s arid Comarca Lagunera, the country’s primary dairy basin, over 450,000 Holstein cows are managed under highly industrialized, technology-intensive schemes [5]. This large-scale, intensive system generates substantial feed bunk refusals, occasionally exceeding 3% of the total mixed ration; however, these feed leftovers retain a high nutritional value [6]. Reclaiming this industrialized dairy waste to supplement less developed, extensive, or semi-extensive small ruminant production systems represents a novel bio-economic paradigm, termed “The Robin Hood Effect” [5,6].

The socio-economic importance of small ruminant production in Mexico has undergone a major structural shift. While the national goat inventory declined by 7.0% between 1970 and 2025, the sheep population expanded by 45%, reaching approximately 8.86 million head [7]. This demographic expansion is particularly pronounced in the arid northern region of the Comarca Lagunera. Within this dairy Holstein basin, hair sheep production has driven a substantial economic spillover; from 2000 to 2025, the regional sheep inventory grew by 250%, culminating in an unprecedented 639% surge in mutton meat production and a 2135% increase in total economic market value [7]. Consequently, optimizing the reproductive efficiency of hair sheep systems in the Comarca Lagunera is crucial to sustaining this accelerated regional growth and maximizing commercial returns under integrated farming schemes. Thus, bridging this growing sheep production with the nutritional leftovers of the Holstein regional industry into more circular, less linear production systems could profoundly enhance the biological, productive, reproductive, and socioeconomic outcomes of small-scale sheep and goat farming [3,5,6].

Phytogenic adaptogens have emerged as strategic tools to optimize reproductive outcomes without synthetic inputs. Two ancestral herbs are particularly interesting. (1) *Withania somnifera* (WITH; Ashwagandha) has robust antioxidant and adaptogenic properties known to mitigate oxidative stress and improve structural sperm parameters [8,9]. (2) *Lepidium meyenii* (LEPI; Maca) is traditionally recognized for its targeted upregulation of copulatory behavior and libido without necessarily altering basal testicular volume [10,11]. The Ashwagandha adaptogen, whose primary compounds are the withanolides, has been shown to modulate the hypothalamic–pituitary–adrenal (HPA) axis to suppress augmented cortisol synthesis, which should reduce the heat stress on developing sperm [8,9]. Regarding Maca, its unique compounds, macamides and macaenes, have been shown to enhance libido and sperm quality, yet independently of cortisol levels [10,11,12].

In addition, both nutraceuticals and adaptogens have generated enhanced productive and reproductive outcomes in previous studies, both in sheep and goats [3,4,5,6]. Critical productive variables—including live weight, metabolic status, sexual odor, scrotal circumference, total testicular volume (TTV), and estimated daily sperm production (EDSP)—peaked in WITH- and LEPI-supplemented rams compared with the control cohort [3]. Thus, using the same sheep breed, husbandry conditions, and location, those findings provide a direct experimental basis for the novel evaluation of sexual drive, seminal quality, and CASA-derived sperm kinematics conducted in the present study. In fact, analyzing the precise velocity profiles, trajectories, and swimming geometry (i.e., sperm kinematics) is certainly indispensable to evaluate the sperm’s functional fertilizing capability [13,14]. Building on these findings, we hypothesized that the strategic administration of these ancestral nutraceuticals (i.e., WITH and LEPI) will optimize biometric traits, testicular size, seminal quality, sexual drive, and computer-assisted sperm analysis (CASA) kinematic traits in Black Belly rams fed under a circular economy feeding approach in dry-semiarid northern Mexico (25° N). Hence, the architectural framework of this study was conceived to elucidate this compelling scientific inquiry.

## 2. Materials and Methods

*General*

Animal care, management, and all experimental protocols strictly followed international [15] and national [16] ethical standards for research welfare. The study adhered to the Mexican Official Standard NOM-062-ZOO-1999 [17] and was formally sanctioned by the institutional research committees under the registration codes UACh-DGIPS-IIBIODEZA 25-020-C-60, 26-056-C-60, and UAAAN-UL-425502002-2832.

### 2.1. Location, Agro-Ecological Features, and Experimental Timeline

Research was carried out at a semi-intensive sheep facility (Ejido Granada, Matamoros, Coahuila, Mexico), situated in the Comarca Lagunera of northern Mexico (25° N, 103° W; altitude: 1110 m). The local climate presents an annual average temperature of 23.8 °C, a yearly rainfall of 230 mm [18], and extreme thermal variations ranging from −1 °C (December/January) to 44 °C (May–June). Executed between May and July, the study overlapped with the peak heat stress period, covering both the pre-breeding transitional phase (May–June) and the actual reproductive season (June–July). During the hotter months of May and June, temperatures regularly peak at 44 °C, while relative humidity averages 30% (min: 17.5%, max: 46%).

### 2.2. Experimental Design, Nutraceutical Dosing, and Dietary Management

Fertile Black Belly rams (n = 15; 72.11 ± 2.4 kg LW; 2.93 ± 0.03 BCS; 4.2 ± 0.4 years old) were randomly assigned to three homogeneous groups (n = 5/each) for a 64-day trial (May–July). Treatments consisted of: (1) CONT (unsupplemented basal diet), (2) WITH (*Withania somnifera* root powder; 5% withanolides; Himalaya Wellness, Sugar Land, Fort Bend, TX, USA), and (3) LEPI (*Lepidium meyenii* root extract; 5% macamides; Nootropics Depot, Portsmouth, Rockingham, NH, USA). Dietary management has been previously described [3]. Briefly, both nutraceuticals were administered each morning orally before feeding at a standardized dose of 400 mg kg^−1^ LW d^−1^, dissolved in 400 mL of distilled water. Individual doses were adjusted weekly based on LW, following preparation protocols by Azeez et al. [19]. The basal diet was an upcycled Total Mixed Ration (TMR) rejection from an intensive Holstein dairy cow operation, evaluated via AOAC methods [20]. On a dry matter (DM) basis (g kg^−1^), the ration comprised corn silage (241.6), rolled corn grain (310.7), alfalfa hay (135.2), soybean meal (123.5), cottonseed meal (94.5), and a mineral–vitamin premix (94.5). Nutritional analysis (%) revealed 80.0 DM, 15.3 crude protein, 26.9 neutral detergent fiber, 17.8 acid detergent fiber, 30.9 starch, 3.8 ether extract, and 7.3 ash, yielding 3.5 Mcal kg^−1^ DM of metabolizable energy and 2.06 Mcal kg^−1^ DM of net energy for lactation. Following a 21-day dietary adaptation period, rams were housed in individual pens and fed twice daily (07:00 and 16:00 h) at 3.5% of their LW, with ad libitum access to water, daily orts removal, and strict sanitary controls.

### 2.3. Animal Biometrics, Sexual Drive, and Computer-Assisted Sperm Kinematics

After the 21-day adaptation period to the basal diet without nutraceutical supplementation, baseline semen collection was performed on Day 0, immediately before the first administration of WITH or LEPI. Treatment administration began immediately thereafter, and subsequent semen collections were performed on Days 32 and 64. Accordingly, biometric and macro-seminal variables were evaluated at three time points: Day 0 (Initial), Day 32 (Middle), and Day 64 (Final). Sexual-drive variables were evaluated according to the schedule described below.

#### 2.3.1. Somatometric Variables

Live weight (LW) was monitored individually using a digital scale with a 150 kg capacity and ±50 g accuracy (WH-C 100, Heyuan City, Guangdong Province, China). Body condition score (BCS) was assessed via lumbar palpation of the spinous and transverse processes on a 1–4 scale (1 = emaciated, 4 = obese) following the standardized methodology of Walkden-Brown et al. [21]. Scrotal circumference (SCRC) was measured at the widest testicular axis using a flexible metric tape graduated in millimeters, according to Cruz-Castrejon et al. [22].

#### 2.3.2. Sexual Drive and Macro-Seminal Assessments

To evaluate the effects of nutraceutical supplementation on sexual behavior and basic ejaculate quality, rams underwent a standardized 15 min libido test. A single trained observer conducted all tests under consistent conditions. Each ram entered an evaluation pen containing a restrained, estrous adult ewe acting as a sexual stimulus for semen collection via an artificial vagina. Timing started upon the ram’s entry. Ejaculation latency (LATEJA, s) was defined as the interval from test start to ejaculation. A successful mount–ejaculation event required mounting, manual penis diversion into the artificial vagina, an ejaculatory thrust, and ejaculate recovery. Rams failing to ejaculate within 15 min were classified as unsuccessful. The mount–ejaculation success rate (MONTEJA, %) was calculated as the proportion of rams succeeding. Ejaculate volume (VOLEJA, mL) was measured directly using graduated collection tubes. The complete sexual-drive and macro-seminal profile included: Sexual Drive–Libido: LATEJA (s) and MONTEJA (%). Macro-Qualitative Semen Traits: VOLEJA (mL), mass motility (MASMOT, units), mass progressive motility (MASPROG, %), sperm concentration (SPERMCON, ×10^6^ mL^−1^), and total sperm number per ejaculate (SPERMCONEJA, ×10^6^ spermatozoa). SPERMCONEJA was calculated as the product of VOLEJA and SPERMCON.

#### 2.3.3. Qualitative Semen Traits and CASA Kinematics

Ejaculates were collected from each ram using an artificial vagina. Semen volume was measured directly in graduated collection tubes, and sperm concentration was determined. Microscopic evaluation was immediately performed to assess macro-qualitative traits, specifically mass motility and mass progressive motility. For objective kinematic track analysis, an aliquot containing 20 *×* 10^6^ spermatozoa was suspended in 245 μL of a sperm motility buffer (150 mM NaCl, 20 mM TES, and 2% seminal plasma) and incubated at 38 °C for 1 min. A 2 μL droplet was loaded onto a pre-warmed (38 °C) glass slide under a 22 *×* 22 mm coverslip. Semen was evaluated using a slide–coverslip preparation instead of a commercial counting chamber. A single trained operator examined five non-overlapping microscopic fields per sample, analyzing at least 200 spermatozoa. Fields with air bubbles, debris, aggregates, or uneven distribution were excluded. These identical selection and exclusion criteria were consistently applied to all samples. Objective motility traits, velocity kinetics, and track geometries were recorded across independent fields using a specialized Sperm Class Analyzer (CASA) system equipped with a digital camera mounted on a BX41 microscope (Olympus, Tokyo, Japan). To ensure a comprehensive evaluation, the complete profile of computer-derived sperm kinematic variables was analyzed, which included the following: (1) *Categorical Motility Traits,* including total motility (TOTMOT, %), progressive motility (PROMOT, %), rapid progressive motility (RAPROMO, %), slow progressive motility (SLOPROMO, %), local mobility (LOCMOB, %), and still spermatozoa (STILL, %); (2) *Velocity Kinetics*, encompassing curvilinear velocity (VCL, μm/s), straight-line velocity (VSL, μm/s), and average path velocity (VAP, μm/s); (3) *Spatial Trajectory Lengths*, including curvilinear distance (DCL, μm), straight-line distance (DSL, μm), and average path distance (DAP, μm); and (4) *Fine-Structure Track Parameters*, namely amplitude of lateral head displacement (ALH, μm), beat-cross frequency (BCF, Hz), head activity (HAC, RAD), oscillation (WOB, %), linearity (LIN, %), and straightness (STR, %).

### 2.4. Statistical Analyses

Before formal hypothesis testing, normality and homoscedasticity of residuals were verified for all continuous variables using the Shapiro–Wilk and Levene’s tests, respectively (PROC UNIVARIATE). Since both nutraceutical supplementation and variable quantification were performed individually, each ram (n = 15) served as the experimental unit. To confirm group homogeneity before the experimental phase, baseline data for animal biometrics (LW, BCS, SCRC), macro-seminal traits (VOLEJA, SPERMCON, SPERMCONEJA, MASMOT, MASPROG), and initial CASA kinematics were analyzed using a one-way ANOVA via the General Linear Models procedure (PROC GLM). The model included the fixed effect of the dietary treatment (CONT, WITH, and LEPI). To assess chronological changes over the 64-day trial under heat stress, continuous traits—including testicular metrics (SCRC), macro-seminal outputs, velocity kinetics (VCL, VSL, VAP), spatial distances (DCL, DSL, DAP), and fine-structure parameters (ALH, BCF, HAC, WOB, LIN, STR)—were analyzed using linear mixed models for repeated measures (PROC MIXED). The model included the fixed effects of treatment (TRT), sampling time (TIME), and their corresponding interaction (TRT *×* TIME). The individual ram nested within the treatment class was specified as the random effect. Since data collection points were equally spaced over time, a first-order autoregressive covariance structure [AR(1)] was applied, which yields mathematically similar results to a spatial power structure [SP(POW)] under equal spacing. To prevent statistical bias caused by the bounded nature of percentages and counts, specialized Generalized Linear Mixed Models (PROC GLIMMIX) were deployed: Categorical sperm motility traits from CASA (TOTMOT, PROMOT, RAPROMO, SLOPROMO, LOCMOB, STILL) and the behavioral success rate (MONTEJA) were analyzed using a beta or binomial distribution with a logit link function. In addition, ejaculation latency (LATEJA) was modeled using a Poisson distribution. When a significant treatment or interaction effect was detected (*p* < 0.05), post hoc pairwise comparisons and multiple-comparison adjustments were performed using the PDIFF/ADJUST = LSD option directly within the LSMEANS statement of the respective procedure (PROC MIXED or PROC GLIMMIX). Statistical significance was declared at *p* < 0.05. All statistical procedures were performed using the SAS software package (SAS Institute Inc., Version 9.4, Cary, NC, USA). All data are presented as Least Squares Means (LSM) ± standard error of the mean.

## 3. Results

### 3.1. Evaluation of Biometric, Testicular, and Sperm–Seminal–Sexual Variables Across Treatments

Biometric, testicular, and sperm–seminal variables across the experimental groups are presented in Table 1. Live weight (LW, 72.11 ± 2.4 kg), body condition score (BCS, 2.98 ± 0.10 units), and scrotal circumference (SCRC, 34.75 ± 1.08 cm) were comparable (*p* > 0.05) among treatments. Similarly, reproductive variables—including latency to ejaculation (LATEJA), volume ejaculated (VOLEJA), and sperm concentration (SPERMCON), did not vary (*p* > 0.05) among treatments, with overall means of 37.79 ± 6.6 s, 0.88 ± 0.15 mL, 3556 ± 198.1 × 10^6^ mL^−1^, and 3090 ± 403.2 × 10^6^ mL^−1^, respectively. Conversely, dietary supplementation altered (*p* < 0.05) mass motility (MASMOT), mass progressive motility (MASPROG), and mount + ejaculation (MONTEJA). Specifically, the WITH group exceeded the CONT group by 332% for MASMOT, 377% for MASPROG, and 200% for MONTEJA. Furthermore, the WITH group outperformed the LEPI group by 24% in MASMOT, 20.1% in MASPROG, and 58% in MONTEJA. In addition, a trend (*p* = 0.07) was observed regarding the total sperm concentration per ejaculate (SPERMCONEJA), which favored the LEPI experimental group.

### 3.2. The Supplementation × Time Interaction Effect on Biometric, Testicular, Behavioral, and Seminal Variables

The analysis of the type of supplementation and the experimental period interaction is presented in Table 2. As observed in the single-effects analysis, no differences (*p* > 0.05) among experimental groups over time occurred regarding body or testicular variables. However, the sexual drive and sperm–seminal variables LATEJA s, VOLEJA mL, SPERMCON, ×10^6^ mL^−1^, SPERMCONEJA, ×10^6^ mL^−1^, MASMOT, units, MASPROG, %, and MONTEJA, %, did differ (*p* < 0.05) due to the treatment × time interaction. In general, the reproductive outcomes of the treatment × time interaction favored (*p* < 0.05) the supplemented groups, which showed the best values in the evaluated responses. Moreover, while the lowest LATEJA (s) was observed in the WITH group (*p* < 0.05), the highest VOLEJA (mL) was observed in the LEPI group (*p* < 0.05). Interestingly, although the highest SPERMCON (×10^6^ mL^−1^) occurred in the supplemented groups (*p* < 0.05), the highest SPERMCONEJA (×10^6^ mL^−1^) was shown by the LEPI group (*p* < 0.05), probably due to a carry-over effect of the higher VOLEJA shown by LEPI.

While the highest sperm motilities, both masal and progressive, were observed in the supplemented groups (*p* < 0.05), the best performance was highlighted in the WITH group. MONTEJA was evaluated only at the initial and final time points. Therefore, intermediate entries in Table 2 are indicated as NE (not evaluated) and do not represent missing observations. Finally, whereas mating + ejaculate (MONTEJA, %) did not occur at the onset of the experimental period, at the end of it, 100% of this sexual behavior, an indirect indicator of libido, was observed in both supplemented groups (WITH and LEPI; *p* < 0.05), with respect to the CONT. Although all the variables analyzed are important, it is central to highlight that the variables LATEJA, MASMOT, and MASPROG showed the highest values (*p* < 0.05) in the *Withania somnifera* group, which additionally showed the highest value for MONTEJA, together with LEPI. Interestingly, this physiological-reproductive scenario occurred without observing differences (*p* > 0.05) among experimental groups with respect to LW, BCS, and SCRC, suggesting a direct influence of the supplemented treatments upon the evaluated responses.

### 3.3. Evaluation of Ram Sperm Kinematics and Trajectory via Computer-Assisted Semen Analysis

The quantitative kinematics of ram spermatozoa evaluated via computer-assisted semen analysis (CASA) are compiled in Table 3. No differences (*p* > 0.05) existed among the experimental groups for any of the categorical motility traits, including total motility (TOTMOT), progressive motility (PROMOT), rapid progressive motility (RAPROMO), slow progressive motility (SLOPROMO), local mobility (LOCMOB), and the static cell fraction (STILL). Similarly, dietary nutraceutical supplementation did not alter (*p* > 0.05) the velocity kinetics—specifically curvilinear velocity (VCL), straight-line velocity (VSL), and average path velocity (VAP)—or their corresponding spatial trajectory lengths, including curvilinear distance (DCL), straight-line distance (DSL), and average path distance (DAP). Furthermore, fine-structure track parameters, such as the amplitude of lateral head displacement (ALH), beat cross frequency (BCF), head activity (HAC), and track oscillation (WOB), remained uniform (*p* > 0.05) across all treatments. In contrast, linearity (LIN) and straightness (STR) were the sole response variables affected (*p* < 0.05) by treatments, with the largest values observed in the supplemented groups (WITH & LEPI). The latter represents an interesting response, as higher LIN and STR values may indicate a more linear and directed sperm-swimming pattern. Such kinematic characteristics could favor sperm progression through the female reproductive tract and increase the likelihood of reaching the oocyte. Undoubtedly, more studies are required to demonstrate direct evidence of fertilizing capacity.

### 3.4. Evaluation of the Longitudinal Trajectory and Treatment × Time Interaction of Sperm CASA-Kinematics

The treatment × time interactions for the CASA-derived kinematic profiles of ram spermatozoa are summarized in Table 4. No interactive effects (*p* > 0.05) were detected among the experimental groups and sampling periods (day 0 vs. day 64) for total, progressive, rapid, or slow motility fractions, nor for velocity parameters (VCL, VSL, VAP), spatial distance metrics (DCL, DSL, DAP), and specific head oscillation traits (ALH, BCF, HAC, WOB). Conversely, significant treatment × time interactions (*p* < 0.05) were observed for local mobility (LOCMOB), linearity (LIN), and straightness (STR). For LOCMOB, the CONT and LEPI groups exhibited a longitudinal decline from the baseline to the end of the 64-day trial. In contrast, the WITH group demonstrated a distinct longitudinal recovery, increasing by 37% over the experimental period. Furthermore, aligning with the main effect outcomes, both LIN and STR exhibited time-dependent increases, reaching their highest values at day 64 in the groups supplemented with either *Withania somnifera* (WITH) or *Lepidium meyenii* (LEPI).

## 4. Discussion

Our findings support the working hypothesis that strategic supplementation with *Withania somnifera* and *Lepidium meyenii* phytogenics optimizes semen quality, sexual drive, and sperm kinematic variables in hair rams fed a basal diet of intensive dairy cattle feed refusals. The primary outcomes confirm that breeding soundness parameters and computer-assisted semen analysis (CASA) kinematic traits varied across treatments over time, consistently favoring the supplemented groups, particularly the WITH cohort. Specifically, the seminal and sexual drive variables MASMOT, MASPROG, and MONTEJA were enhanced by both supplements, while WITH outperformed all other groups; a marginal trend (*p* = 0.07) was observed for SPERMCONEJA favoring LEPI. Furthermore, a significant treatment × time interaction occurred for the kinematic indexes LOCMOB, LIN, and STR; although WITH and LEPI yielded comparable results, both significantly surpassed the CONT group. Remarkably, these physiological and kinetic improvements were achieved in Black Belly rams consuming an agro-industrial waste-based diet, demonstrating that nutritional supplementation mitigates the severe environmental heat stress characteristic of the study region during the summer months (May–July) (Figure 1). Undoubtedly, the energy-dense, low-fiber nature of this Holstein TMR refusal (3.5 Mcal kg^−1^ DM ME; 26.9% NDF) shapes the physiological rationale of this trial. Pairing this surplus with WITH/LEPI, rather than a purpose-formulated, fiber-balanced ration, is precisely what anchors the circular-economy relevance of upcycling dairy-industry byproducts for small ruminant systems. Because basal diet composition can influence reproductive responses, the effects observed in this study may differ in magnitude when WITH and LEPI are used with other dietary formulations.

### 4.1. Potential Mechanisms Driving Productive and Reproductive Improvements

These research outcomes are particularly central given that the evaluated response variables are key to both the productive and reproductive performance of small ruminants. Consistently, distinct variations in corporal, productive, and reproductive traits following herbal supplementation have been documented across various ovine studies [23,24,25,26,27]. In the present study, the beneficial effects observed in the WITH cohort should likely be driven by its primary bioactive compounds, mainly withanolides, sitoindosides, and alkaloids, which are known to enhance growth and modulate anti-stress, antioxidant, immunomodulatory, and hepatoprotective pathways [28,29,30,31]. Furthermore, the superior performance of the LEPI group relative to the CONT group may be attributed to the action of a complex matrix of bioactive secondary metabolites such as macamides, macaenes, glucosinolates, and alkaloids, involved in the systemic regulation of energy metabolism, thermoregulation, and electrolyte balance, alongside its capacity to enhance physical stamina and mitigate thermal stress [32,33], aligned with its documented thermoregulatory action in mammals [33,34]. Collectively, our findings demonstrate that time-dependent supplementation with WITH and LEPI significantly improves productive and reproductive parameters in Black Belly rams consuming an agro-industrial waste-based diet composed of dairy cattle feed refusals. Nevertheless, the precise physiological mechanisms and specific anatomical sites of action of these phytogenics on corporal, sexual drive, and reproductive traits remain to be fully elucidated; the need for future molecular and systemic endocrine research is undoubtedly a pending assignment.

### 4.2. Influence of Herbal Nutraceuticals on Libido and Breeding Soundness Parameters

The significant increase in sexual drive and qualitative seminal parameters observed in the supplemented groups—specifically regarding MASMOT, MASPROG, and the mounting behavior (MONTEJA)—highlights the targeted reproductive support offered by the phytogenic supplements WITH and LEPI. The marked superiority of the *Withania somnifera* group in sexual drive and general semen traits could plausibly be related to its reported neuroendocrine modulating capacity. In males, WITH-oral administration has been reported to induce a robust systemic elevation in the blood concentrations of both free testosterone and luteinizing hormone (LH) [35]. At the physiological level, WITH actively improves prosexual and sexual behavior in sexually stressed subjects by increasing non-contact erections, mounts, intromission, and ejaculation frequencies, while decreasing sexual exhaustion through the shortening of post-ejaculatory intervals and latencies [36]. Crucially, the WITH-adaptogen has been reported to modulate central neurotransmitters and hormones associated with sexual desire and stress—including dopamine, serotonin, corticosterone, and prolactin—while promoting a dose-dependent increase in serum LH, FSH, and testosterone levels [36]. Structural and biochemical adaptations described in other models may support these behavioral improvements; WITH has been shown, in other species, to enhance penile histomorphology and upregulate the expression of essential biomarkers associated with penile erection facilitation, such as Neuronal Nitric Oxide Synthase, Endothelial Nitric Oxide Synthase, and the activated form of Protein Kinase B, Nitric Oxide, Acetylcholine, and cGMP, yielding therapeutic outcomes comparable to sildenafil [36].

Concurrently, the positive impacts of *Lepidium meyenii* (LEPI) on libido and sperm concentration per ejaculate (SPERMCONEJA) match previous evidence detailing its positive role in overhauling male fecundity. Longitudinal assessments of LEPI supplementation during the reproductive season from April to July have revealed a progressive and significant increase in sperm concentrations, total sperm count, and ejaculated volume over a 90-day trial [37], validating similar endocrinological behaviors reported in rodents [38] and humans [39]. Although some human models using Maca supplementation over 12 weeks did not promote differences in semen volume, sperm motility, or normal morphology, they established a significant and targeted increase in raw sperm concentration compared to control cohorts [40]. In scenarios of severe environmental heat stress, oral administration of Maca has been reported to preserve breeding soundness by preventing heat-induced testicular degeneration, maximizing growth performance, feed intake, and feed conversion ratios, while downregulating systemic cortisol and boosting endogenous testosterone synthesis, libido, total antioxidant capacity, and superoxide dismutase levels [41]. This multidimensional support aligns with recent models in domestic mammals, in which dietary Maca led to robust upregulation of ejaculate volume, total sperm count, morphology, and progressive motility in both normofertile and subfertile individuals [42]. A similar systemic hormone-restoration mechanism could plausibly contribute to why the nutraceutical cohorts in this trial significantly outperformed the CONT group.

### 4.3. Impact of Supplementation on Quantitative Sperm Kinematics via CASA

As previously reported, while conventional semen analysis primarily evaluates sperm concentration and motility, the computer-assisted semen analysis (CASA) enables a more in-depth quantification of sperm motility parameters, involving velocity, path length, and directionality [13,14]. Our study revealed a significant treatment × time (TRT × TIME) interaction for local mobility (LOCMOB), linearity (LIN), and straightness (STR), demonstrating that the kinetic profile of ram spermatozoa undergoes a time-dependent optimization under phytogenic influence. Mechanistically, this kinetic acceleration is intimately tied to the preservation of sperm structural integrity against environmental insults. In fact, increased sperm DNA fragmentation has been associated with impaired sperm motility efficiency and directionality, rather than a uniform reduction in motility [43]. Moreover, in ex vivo conservation models, the addition of Maca to semen extenders has been shown to preserve sperm DNA and plasma membrane integrity, decrease lipid peroxidation, and enhance curvilinear velocity and hyperactivation traits over prolonged storage [44]. Similarly, under chronic environmental challenge, oral supplementation with Maca has been reported to mitigate oxidative damage by maximizing total antioxidant capacity and superoxide dismutase levels while minimizing malondialdehyde concentrations, exhibiting improvements in sperm motility, survival, membrane functionality, and concentration, with a decrease in abnormal sperm values in that model [41]. At the cellular and subcellular level, this defense has been described to shield the plasma membrane and acrosomes, helping maintain a normal structure and well-organized axonemal components within the tail in that same report [41].

The fact that linearity LIN and STR depicted increased values in the WITH and LEPI groups provides physiological insights potentially related to energy conservation and cervical mucus navigation. Such an increase suggests that spermatozoa may be swimming in a cleaner, straighter trajectory rather than wasting metabolic energy on excessive lateral head displacement (ALH) or frantic winding paths (VCL), consistent with reports that high linearity and straightness profiles are associated with efficient forward trajectory and cervical mucus penetration in other studies [45]. However, as CASA kinematics are associative proxy traits, this study did not directly assess fertilizing capacity, the latter being an unquestionable pending scientific assignment. The longitudinal response of LOCMOB, LIN, and STR is biologically consistent with the duration of the intervention. A complete spermatogenic cycle in rams lasts approximately 47–49 days, followed by epididymal maturation [46]. Therefore, during the 64-day trial, the spermatozoa collected at the final evaluation developed under sustained nutraceutical exposure. This biological timeline may explain the absence of initial differences and the targeted kinematic improvements observed at day 64. Regarding LOCMOB, it often represents sperm that are alive and metabolically active but vibrating or moving in place rather than swimming forward. The longitudinal decline observed in the CONT group, potentially associated with the severe environmental heat stress or photoperiod of May–July, contrasted with the 37% increase observed in the WITH group. This contrasting response is consistent with a potential adaptogenic and antioxidant effect of *Withania somnifera*, whose active components may help preserve the structural integrity and mitochondrial machinery of developing spermatozoa against heat-induced oxidative stress [1,8]. Concurrently, the synchronized increase in LIN and STR on day 64 under both WITH and LEPI supplementation suggests that these phytogenics may contribute to axonemal symmetry or ATP distribution during epididymal maturation, favoring a straighter, more efficient swimming pattern. The mechanisms and sites of action of the supplemented nutraceuticals (i.e., WITH and LEPI) on sexual drive, seminal quality, and sperm kinematics cannot be fully elucidated without complementary research, particularly at the physiological and molecular levels. Although a larger sample size would strengthen the inferences, the strict phenotypic homogeneity of the experimental units and the repeated-measures design allowed for the identification of clear biological trends. Undoubtedly, the proposed neuroendocrine and antioxidant mechanisms remain biologically plausible interpretations rather than directly demonstrated pathways. In fact, these findings provide an initial physiological and kinematic characterization that justifies future trials integrating endocrine, oxidative, and in vivo fertility assessments.

### 4.4. Beyond the Microscope: Sperm Kinetics and Upregulation of the Livestock Circular Economy (Bovine-Ovine) in Northern Arid Mexico

By breaking the orthodox isolation of reproductive physiology, our findings connect cellular metrics with systemic territorial resilience. In the Comarca Lagunera, a region suffocated by water scarcity and hyper-industrialized bovine dairy monopolies [47,48], identifying rams with superior sperm kinematic efficiency, optimal traditional seminal parameters, and an unyielding sexual drive ceases to be a mere biological finding; it becomes the technical guarantee to secure a decentralized “Robin Hood Effect”. When 4.5% of daily feed refusal from the Holstein industry—around 265,000 milking Holsteins [7]—is upcycled into a low-cost nutritional matrix for less linear-industrialized livestock production systems [3,4,6,23], reproductive efficiency becomes the critical variable. The central idea is to generate high-performing animals to maximize the metabolic conversion of those 215.4 tons of daily dry-matter surplus into not only demographic growth but also reproductive-productive efficiency [3,4,6,23]. Accelerating the expansion of the ovine census to 180,000 heads [23] requires maximizing pregnancy rates per service through precise sperm kinetics. Consequently, the laboratory microscope and CASA device shift from an elite, research-academic, centralized tool towards a democratic-horizontal instrument, translating cellular velocity into 25.10 million USD of regional economic output [49] while safeguarding the food sovereignty of more than 20,000 vulnerable households [23] under the 2030 Agenda [50]. This bio-economic approach directly translates microscopic cellular movements—such as track linearity and straightness—into broad macro-agricultural metrics. Ultimately, *“The Robin Hood Effect”* should be interpreted as a conceptual bio-economic framework linking feed upcycling, reproductive efficiency, and regional resilience, rather than as a directly measured socioeconomic outcome. Although the biological findings support this system-level potential, farm-level costs, profitability, household income, and technology adoption were not evaluated and require future bio-economic assessment; some interesting bio-economic trends have been previously addressed [3,5,6,23].

## 5. Conclusions

Strategic *Withania somnifera* (WITH) and *Lepidium meyenii* (LEPI) phytogenic supplementation successfully mitigates the depressive effects of seasonal summer heat stress on the reproductive performance of Black Belly rams in northern arid Mexico. Rather than inducing a state of metabolic exhaustion or premature hyperactivation, the supplemented adaptogens optimize quantitative sperm kinematics—specifically increasing track linearity and straightness—while concurrently enhancing libido, mounting behavior, and traditional breeding soundness metrics. These synchronized physiological improvements validate the biological timeline of a complete spermatogenic cycle, demonstrating that the full therapeutic potential of these nutraceuticals is achieved during long-term chronological exposure. Crucially, this study demonstrates that high-precision reproductive diagnostics (i.e., CASA) can be seamlessly coupled with low-cost, non-linear circular production systems. Achieving superior sperm kinematic efficiency in animals fed a basal diet derived from industrial dairy feed refusals proves that reproductive efficiency is the ultimate catalyst needed to scale upcycled nutritional matrices, the so-called “Robin Hood Effect”. Optimizing the reproductive output per sire transforms advanced laboratory diagnostics from an isolated academic-research exercise into a horizontal socio-economic tool capable of driving sustainable, decentralized livestock growth and strengthening household resilience in climatically, hydric, and socio-economically vulnerable arid territories. The latter is undoubtedly central not only for the enhancement of small-scale small ruminant schemes but also for the livelihoods of sheepherders and their families.

## Figures and Tables

**Figure 1:**
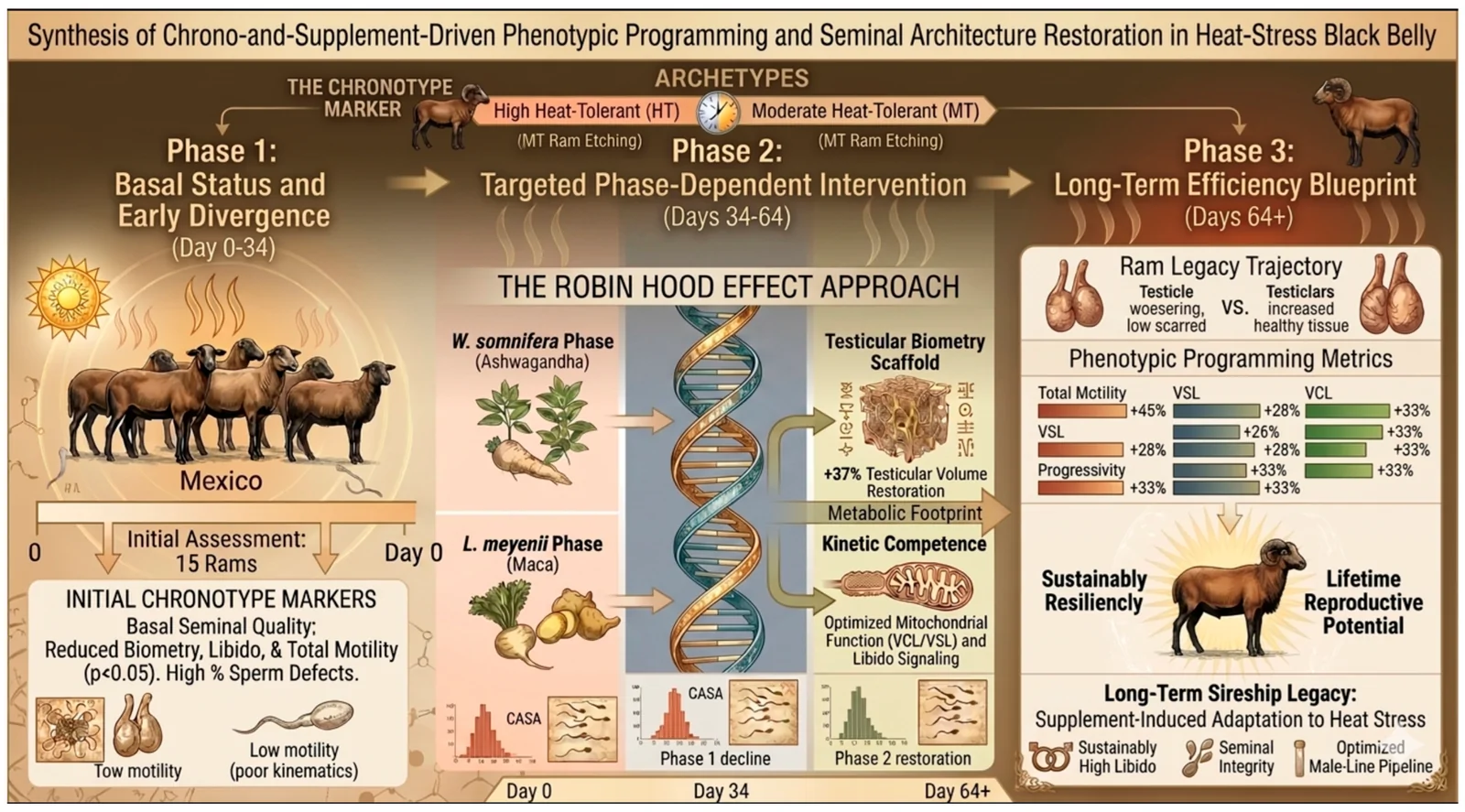
Conceptual synthesis diagram integrating the experimental design, principal outcomes, and proposed biological interpretation of the inter-systemic bio-circular framework defined as *“The Robin Hood Effect Approach,”* in which nutrient-dense feed refusals from an intensive dairy-cattle operation are upcycled to support a vulnerable smallholder small-ruminant production system during severe summer heat stress (May–July; 25° N, arid northern Mexico). The longitudinal timeline depicts a 64-day experimental period encompassing a complete spermatogenic cycle across three cohorts (n = 5 per group): non-supplemented control (CONT), *Withania somnifera* (WITH; 400 mg kg^−1^ LW d^−1^), and *Lepidium meyenii* (LEPI; 400 mg kg^−1^ LW d^−1^). *Phase 1 (Basal Status and Early Divergence)* establishes the baseline reproductive status under the environmental heat challenge. *Phase 2 (Targeted Phase-Dependent Intervention)* represents the progressive action of adaptogenic nutraceutical supplementation on reproductive and sperm kinematic responses. *Phase 3 (Long-Term Efficiency Blueprint)* displays the final phenotypic and kinematic outcomes at day 64. Chronological exposure to WITH generated a *37% relative increase in local mobility (LOCMOB), from 1.47% at the initial evaluation to 2.01% at the final evaluation*, while both WITH and LEPI improved CASA-derived sperm trajectory geometry, particularly straightness (STR) and linearity (LIN). This horizontal integration highlights how agro-industrial feed refusals can support reproductive efficiency and strengthen the sustainability and climate resilience of smallholder farming systems under hyperarid conditions.

**Table 1:** Least squares mean (± SEM) for biometric, testicular, sexual-drive, and seminal variables in Black Belly rams (n = 15) fed a basal diet of upcycled Holstein ration rejections and either non-supplemented (CONT) or supplemented with *Withania somnifera* or *Lepidium meyenii* in northern arid Mexico (25° N) ^1^.

Variable ^2^	CONT	WITH	LEPI	SEM ^3^	*p*-Value
LW (kg)	72.76 ^a^	71.44 ^a^	72.15 ^a^	2.44	0.19
BCS (units)	3.0 ^a^	2.95 ^a^	3.0 ^a^	0.05	0.42
SCRC (cm)	34.76 ^a^	34.80 ^a^	34.69 ^a^	1.08	0.51
LATEJA (s)	40.33 ^a^	32.85 ^a^	40.19 ^a^	6.68	0.54
VOLEJA (mL)	0.87 ^a^	0.67 ^a^	1.10 ^a^	0.15	0.18
SPERMCON (×10^6^ mL^−1^)	3338 ^a^	3706 ^a^	3624 ^a^	198	0.50
SPERMCONEJA(×10^6^ mL^−1^)	2903 ^a^	2487 ^a^	3985 ^a^	503	0.07
MASMOT (%)	0.93 ^b^	3.09 ^a^	2.48 ^ab^	0.69	0.05
MASPROG (%)	16.6 ^b^	62.7 ^a^	52.2 ^a^	15.77	0.05
MONTEJA (%)	33.0 ^b^	66.0 ^a^	41.6 ^ab^	0.08	0.05

^1^ *Withania somnifera* (WITH; 400 mg kg^−1^ LW d^−1^), or *Lepidium meyenii* (LEPI; 400 mg kg^−1^ LW d^−1^) during a 64 d experimental period (May–July). ^2^ Response variables included: live weight (LW, kg), body condition score (BCS, units), scrotal circumference (SCRC, cm), latency to ejaculation (LATEJA, s), volume ejaculated (VOLEJA, mL), sperm concentration (SPERMCON, ×10^6^ mL^−1^), sperm concentration per ejaculate (SPERMCONEJA, ×10^6^ mL^−1^), mass motility (MASMOT, units), mass progressive (MASPROG%), and mount + ejaculation (MONTEJA, %). ^3^ The most conservative standard error of the least squares means (SEM) is presented. ^a–b^ Least squares means with different superscripts within variables among treatments differ (*p* < 0.05).

**Table 2:** Least squares means for the longitudinal treatment × time interaction regarding biometric, testicular, sexual-drive, and seminal variables in Black Belly rams (n = 15) fed a basal diet of upcycled Holstein ration rejections and either non-supplemented (CONT) or supplemented with *Withania somnifera* or *Lepidium meyenii* in northern arid Mexico (25° N) ^1^.

Variable ^2^	CONT				WITH			LEPI		*p*-Value
	Initial	Middle	Final	Initial	Middle	Final	Initial	Middle	Final	
LW	73.4 ^a^	72.8 ^a^	72.2 ^a^	71.1 ^a^	71.1 ^a^	72.1 ^a^	71.7 ^a^	72.1 ^a^	72.6 ^a^	0.22
BCS	3.0 ^a^	3.0 ^a^	3.0 ^a^	2.8 ^b^	3.0 ^a^	3.0 ^a^	3.0 ^a^	3.0 ^a^	3.0 ^a^	0.62
SCRC	34.3 ^a^	34.0 ^a^	33.6 ^a^	34.5 ^a^	34.8 ^a^	35.0 ^a^	34.1 ^a^	33.5 ^a^	34.0 ^a^	0.06
LATEJA	48.3 ^a^	35.5 ^bc^	50.3 ^a^	31.5 ^bc^	28.1 ^cd^	26.7 ^d^	44.7 ^ab^	59.5 ^a^	24.3 ^a^	0.03
VOLEJA	0.9 ^a^	1.1 ^ab^	0.7 ^c^	0.5 ^c^	0.9 ^a^	0.6 ^c^	1.2 ^a^	1.2 ^a^	0.8 ^bc^	0.03
SPERMCON	3256 ^b^	3121 ^b^	3421 ^ab^	3448 ^a^	3707 ^a^	3971 ^a^	3635 ^a^	3448 ^a^	3520 ^a^	0.05
SPERMCONEJA	2930 ^b^	3427 ^b^	2396 ^c^	1696 ^c^	3316 ^a^	2377 ^c^	4300 ^a^	4135 ^a^	3020 ^b^	0.04
MASMOT	0.7 ^c^	0.7 ^c^	0.7 ^c^	1.5 ^ab^	3.6 ^a^	3.7 ^a^	2.0 ^a^	1.7 ^ab^	2.7 ^a^	0.05
MASPROG	10.0 ^c^	10.0 ^c^	20.1 ^c^	25.1 ^b^	79.1 ^a^	73.7 ^a^	38.3 ^b^	31.2 ^b^	56.7 ^a^	0.04
MONTEJA	0.0 ^b^	NE	40 ^b^	0.0 ^b^	NE	100 ^a^	0.0 ^b^	NE	100 ^a^	0.01

^1^ *Withania somnifera* (WITH; 400 mg kg^−1^ LW d^−1^), or *Lepidium meyenii* (LEPI; 400 mg kg^−1^ LW d^−1^) during a 64 d experimental period (May–July). Initial, Middle, and Final correspond to Day 0, Day 32, and Day 64 of the trial, respectively. ^2^ Response variables involved: live weight (LW, kg), body condition score (BCS, units), scrotal circumference (SCRC, cm), latency to ejaculation (LATEJA, s), volume ejaculated (VOLEJA, mL), sperm concentration (SPERMCON, ×10^6^ mL^−1^), sperm concentration per ejaculate (SPERMCONEJA, ×10^6^ mL^−1^), mass motility (MASMOT, units), mass progressive motility (MASPROG, %), and mount + ejaculation (MONTEJA, %). ^2^ Phase of the experimental period (May–July). ^a–d^ Least squares means with different superscripts within variables among treatments differ (*p* < 0.05). NE, not evaluated.

**Table 3:** Least squares means for computer-assisted semen analysis (CASA) sperm kinematic parameters in Black Belly rams (n = 15) fed a basal diet of upcycled Holstein ration rejections and either non-supplemented (CONT) or supplemented with *Withania somnifera* or *Lepidium meyenii* in northern arid Mexico (25° N) ^1^.

Variable ^2^	CONT	WITH	LEPI	SEM ^3^	*p*-Value
TOTMOT (%)	46.77 ^a^	59.6 ^a^	38.62 ^a^	15.1	0.67
PROMOT (%)	45.00 ^a^	57.93 ^a^	34.78 ^a^	15.9	0.64
RAPROMO (%)	27.22 ^a^	36.82 ^a^	21.57 ^a^	11.8	0.64
SLOPROMO (%)	17.78 ^a^	21.13 ^a^	13.4 ^a^	5.3	0.65
LOCMOB (%)	1.75 ^a^	1.65 ^a^	3.83 ^a^	1.02	0.32
STILL (%)	53.23 ^a^	40.42 ^a^	61.40 ^a^	15.1	0.62
VCL (μm/s)	59.08 ^a^	77.02 ^a^	47.75 ^a^	19.2	0.58
VSL (%)	25.67 ^a^	30.23 ^a^	22.07 ^a^	7.9	0.73
VAP (μm/s)	30.88 ^a^	39.07 ^a^	25.98 ^a^	9.3	0.61
DCL	19.93 ^a^	25.15 ^a^	15.63 ^a^	6.3	0.60
DSL	8.07 ^a^	9.13 ^a^	6.62 ^a^	2.6	0.77
DAP	9.93 ^a^	12.22 ^a^	7.98 ^a^	3.1	0.64
ALH (μm)	0.77 ^a^	1.03 ^a^	0.65 ^a^	0.24	0.55
BCF (Hz)	7.22 ^a^	7.72 ^a^	6.07 ^a^	2.35	0.84
HAC (RAD)	0.18 ^a^	0.23 ^a^	0.13 ^a^	0.06	0.37
WOB (%)	0.57 ^a^	0.53 ^a^	0.60 ^a^	0.04	0.58
LIN (%)	0.47 ^b^	0.62 ^a^	0.54 ^a^	0.03	0.05
STR (%)	0.77 ^b^	0.88 ^a^	0.85 ^a^	0.01	0.01

^1^ *Withania somnifera* (WITH; 400 mg kg^−1^ LW d^−1^), or *Lepidium meyenii* (LEPI; 400 mg kg^−1^ LW d^−1^) during a 64 d experimental period (May–July). ^2^ Kinematic parameters involved: total motility (TOTMOT, %), progressive motility (PROMOT, %) rapid progressive motility (RAPROMO, %), slow progressive motility (SLOPROMO, %), local mobility (LOCMOB, %), still (STILL, %), velocity curved line (VCL, μm/s), velocity straight line (VSL, μm/s), velocity average path (VAP, μm/s), distance curvilinear (DCL, μm), distance straight linear (DSL, μm), distance average path (DAP, μm), amplitude of lateral head (ALH, μm), beat cross frequency (BCF, Hz), head activity (HAC, RAD), oscillation (WOB, %), linearity (LIN, %) and straightness (STR,%). ^3^ The most conservative standard error of the least squares means is presented. ^a–b^ Least squares means with different superscripts within variables among treatments differ (*p* < 0.05).

**Table 4:** Least squares means for the treatment × time interaction for computer-assisted semen analysis (CASA) sperm kinematic parameters in Black Belly rams (n = 15) fed a basal diet of upcycled Holstein ration rejections and either non-supplemented (CONT) or supplemented with *Withania somnifera* or *Lepidium meyenii* in northern arid Mexico (25° N) ^1^.

Variable ^2^	CONT		WITH		LEPI		*p*-Value
	Initial	Final	Initial	Final	Initial	Final	
TOTMOT (%)	66.57 ^a^	26.97 ^a^	54.77 ^a^	64.43 ^a^	25.17 ^a^	52.07 ^a^	0.49
PROMOT (%)	64.10 ^a^	25.90 ^a^	53.27 ^a^	62.60 ^a^	19.13 ^a^	50.43 ^a^	0.46
RAPROMO (%)	37.80 ^a^	16.63 ^a^	35.00 ^a^	38.63 ^a^	5.93 ^a^	37.20 ^a^	0.43
SLOPROMO (%)	26.30 ^a^	9.27 ^a^	18.30 ^a^	23.97 ^a^	13.20 ^a^	13.60 ^a^	0.48
LOCMOB (%)	2.47 ^b^	1.03 ^b^	1.47 ^b^	2.01 ^b^	3.03 ^a^	2.63 ^b^	0.05
STILL (%)	33.43 ^a^	73.03 ^a^	45.27 ^a^	35.57 ^a^	74.83 ^a^	47.97 ^a^	0.55
VCL (μm/s)	77.70 ^a^	40.47 ^a^	74.73 ^a^	79.30 ^a^	26.90 ^a^	68.60 ^a^	0.49
VSL (μm/s)	32.10 ^a^	19.23 ^a^	30.07 ^a^	30.40 ^a^	12.03 ^a^	32.10 ^a^	0.59
VAP (μm/s)	39.10 ^a^	22.67 ^a^	37.80 ^a^	40.33 ^a^	15.27 ^a^	36.70 ^a^	0.53
DCL (μm)	27.70 ^a^	12.17 ^a^	24.20 ^a^	26.10 ^a^	8.33 ^a^	22.93 ^a^	0.44
DSL (μm)	11.03 ^a^	5.10 ^a^	9.07 ^a^	9.20 ^a^	3.10 ^a^	10.13 ^a^	0.55
DAP (μm)	13.57 ^a^	6.30 ^a^	11.73 ^a^	12.70 ^a^	4.23 ^a^	11.73 ^a^	0.49
ALH (μm)	1.07 ^a^	0.47 ^a^	1.00 ^a^	1.07 ^a^	0.40 ^a^	0.90 ^a^	0.41
BCF (Hz)	9.87 ^a^	4.57 ^a^	7.33 ^a^	8.10 ^a^	3.20 ^a^	8.93 ^a^	0.60
HAC (RAD)	0.23 ^a^	0.13 ^a^	0.23 ^a^	0.23 ^a^	0.07 ^a^	0.20 ^a^	0.40
WOB (%)	0.50 ^a^	0.63 ^a^	0.57 ^a^	0.50 ^a^	0.63 ^a^	0.57 ^a^	0.37
LIN (%)	0.40 ^c^	0.53 ^b^	0.53 ^b^	0.62 ^a^	0.50 ^ab^	0.56 ^a^	0.01
STR (%)	0.78 ^b^	0.72 ^b^	0.82 ^a^	0.86 ^a^	0.83 ^a^	0.87 ^a^	0.03

^1^ *Withania somnifera* (WITH; 400 mg kg^−1^ LW d^−1^) or *Lepidium meyenii* (LEPI; 400 mg kg^−1^ LW d^−1^) during a 64 d experimental period (May–July). ^2^ Kinematic parameters involved: total motility (TOTMOT, %), progressive motility (PROMOT, %) rapid progressive motility (RAPROMO, %), slow progressive motility (SLOPROMO, %), local mobility (LOCMOB, %), still (STILL, %), velocity curved line (VCL, μm/s), velocity straight line (VSL, μm/s), velocity average path (VAP, μm/s), distance curvilinear (DCL, μm), distance straight linear (DSL, μm), distance average path (DAP, μm), amplitude of lateral head (ALH, μm), beat cross frequency (BCF, Hz), head activity (HAC, RAD), oscillation (WOB, %), linearity (LIN, %), and straightness (STR,%). ^a–c^ Least squares means with different superscripts within variables among treatments differ (*p* < 0.05).

## Data Availability

The original contributions presented in this study are included in the article. Further inquiries can be directed to the corresponding authors.

## Abbreviations

The following abbreviations are used in this manuscript:

ALH	Amplitude lateral head
ANOVA	Analysis of variance
AOAC	Association of Official Analytical Chemists
ATP	Adenosine triphosphate
BCS	Body condition score
BCF	Beat cross frequency
CASA	Computer-assisted sperm analysis
cGMP	Cyclic guanosine monophosphate
CONT	Non-supplemented
DAP	Distance average path
DCL	Distance curvilinear
DM	Dry matter
DMI	Dry matter intake
DNA	Deoxyribonucleic acid
DSL	Distance straight linear
EDSP	Estimated daily sperm production
FAO	Food and Agriculture Organization of the United Nations
FSH	Follicle-stimulating hormone
HAC	Head activity
HPA	Hypothalamic–pituitary–adrenal
LATEJA	Latency to ejaculation
LEPI	*Lepidium meyenii*
LH	Luteinizing hormone
LIN	Linearity
LOCMOB	Local mobility
LW	Live weight
MASMOT	Mass motility
MASPROG	Mass progressive motility
MC	Milked cow
MONTEJA	Mount–ejaculation success rate
MUSD	Millions of United States dollars
NEL	Net Energy for Lactation
ODOR	Sexual odor
PRO	Serum protein
PROMOT	Progressive motility
RAPROMO	Rapid progressive motility
SC	Scrotal circumference
SEM	Standard error of the mean
SLOPROMO	Slow progressive motility
SPERMCON	Sperm concentration
SPERMCONEJA	Total sperm number per ejaculate
STILL	Immotile spermatozoa
STR	Straightness
TMR	Total Mixed Ration
TOTMOT	Total motility
TRT	Treatment
VAP	Velocity average path
VCL	Velocity curved line
VOLEJA	Total ejaculate volume
VSL	Velocity straight line
WITH	*Withania somnifera*
WOB	Oscillation
